# The combined effect of graphene oxide and elemental nano-sulfur on soil biological properties and lettuce plant biomass

**DOI:** 10.3389/fpls.2023.1057133

**Published:** 2023-03-14

**Authors:** Tereza Hammerschmiedt, Jiri Holatko, Radim Zelinka, Antonin Kintl, Petr Skarpa, Zuzana Bytesnikova, Lukas Richtera, Adnan Mustafa, Ondrej Malicek, Martin Brtnicky

**Affiliations:** ^1^ Department of Agrochemistry, Soil Science, Microbiology and Plant Nutrition, Faculty of AgriSciences, Mendel University in Brno, Brno, Czechia; ^2^ Agrovyzkum Rapotin, Ltd., Rapotin, Czechia; ^3^ Department of Chemistry and Biochemistry, Mendel University in Brno, Brno, Czechia; ^4^ Agricultural Research, Ltd., Troubsko, Czechia; ^5^ Institute of Chemistry and Technology of Environmental Protection, Faculty of Chemistry, Brno University of Technology, Brno, Czechia; ^6^ Institute for Environmental Studies, Faculty of Science, Charles University in Prague, Praha, Czechia

**Keywords:** soil amendments, agricultural production, microbial activity, nutrient cycling, sulfur nutrition

## Abstract

The impact of graphene oxide (GO) nanocarbon on soil properties is mixed, with both negative and positive effects. Although it decreases the viability of some microbes, there are few studies on how its single amendment to soil or in combination with nanosized sulfur benefits soil microorganisms and nutrient transformation. Therefore, an eight-week pot experiment was carried out under controlled conditions (growth chamber with artificial light) in soil seeded with lettuce (*Lactuca sativa*) and amended with GO or nano-sulfur on their own or their several combinations. The following variants were tested: (I) Control, (II) GO, (III) Low nano-S + GO, (IV) High nano-S + GO, (V) Low nano-S, (VI) High nano-S. Results revealed no significant differences in soil pH, dry plant aboveground, and root biomass among all five amended variants and the control group. The greatest positive effect on soil respiration was observed when GO was used alone, and this effect remained significant even when it was combined with high nano-S. Low nano-S plus a GO dose negatively affected some of the soil respiration types: NAG_SIR, Tre_SIR, Ala_SIR, and Arg_SIR. Single GO application was found to enhance arylsulfatase activity, while the combination of high nano-S and GO not only enhanced arylsulfatase but also urease and phosphatase activity in the soil. The elemental nano-S probably counteracted the GO-mediated effect on organic carbon oxidation. We partially proved the hypothesis that GO-enhanced nano-S oxidation increases phosphatase activity.

## 1 Introduction

In the last decade, 2D carbon-based nanomaterials, such as graphene, graphene oxide, and reduced graphene oxide have been widely applied in various experimental and technological fields, primarily to purify aquatic and soil environments from pollutants. (Teng et al., 2019). GO (42-62% wt carbon, 24-36% wt oxygen) can be prepared from graphite in the laboratory using the Hummers and Offemans method (Marcano et al., 2010; Yu et al., 2016), and further modified by reduction of C=O groups or their derivatization e.g., with metal atoms (Zn, Cu, Ag), which enables environmental adsorptive detoxication from metalloids (Zhang et al., 2020; Sengupta et al., 2022). GO is quite mobile in soil (Sangani et al., 2019; Xia et al., 2021); however, its leaching can be reduced by aggregation mediated by Ca^2+^ in concentration ≥ 0.5 mM (Qi et al., 2014). Amendment of GO to soil alters its hygroscopic and adsorptive capabilities and water content, and reduces the impact of drought stress (Zhao et al., 2020; Zhao et al., 2022); as a carrier, it increases the uptake of mineral micronutrients to plants *via* controlled release (Kabiri et al., 2017; Li et al., 2019; Carneiro et al., 2022; Mohammadi Alagoz et al., 2022). These effects on soil nutritional traits are positive for plant growth and physiology (Lahiani et al., 2015; Juarez-Maldonado et al., 2019) and GO may also benefit by protecting against other plant-harming factors (Arikan et al., 2022). However, the impact of GO on plants is dose-dependent, and high levels of application (up to 2000 mg/L) have been found to lead to increased reactive oxygen species in cabbage, necrotic lesions in tomatoes, and decreased photosystem II activity in peas. (Samadi et al., 2021).

GO has also been found to exhibit varying levels of toxicity towards bacteria, fungi, and algae, negatively impacting their growth and altering the structure of microbial communities in soil (Gurunathan et al., 2012; Chung et al., 2015; Du et al., 2015; Forstner et al., 2019). GOs are generally more toxic to gram-positive bacteria (Kulshrestha et al., 2017) but showed neutral to positive effects on other soil microbes (Wang et al., 2003; Ge et al., 2016). It was also reported that GOs contain soil biological properties (Ren et al., 2015). In some cases, GO has been found to increase bacterial community richness in a concentration-dependent manner (Luo et al., 2022). It has been acknowledged that the integration of GO and other nanomaterials may improve GO properties (Yap et al., 2019; Hammerschmiedt et al., 2022) and provide new specifically-featured materials (Gupta et al., 2015; Liu et al., 2017), which may ameliorate salinity stress on crops (Zahedi et al., 2023). In this context, one of the promising types of nanomaterials is nanosized elemental sulfur. It is highly beneficial in agriculture (Teng et al., 2019) because it is insoluble and thus, does not leach after being added to soil (Riley et al., 2002; Lee et al., 2011; Lucheta and Lambais, 2012; Samadi et al., 2021), and it promotes tolerance to abiotic and biotic stresses in plants (Fuentes-Lara et al., 2019). It should be noted that elemental sulfur needs to be oxidized by bacteria into sulfates ( ${\text{SO}}_{4}^{2-}$ ) in order to serve as a nutrient for soil organisms and plants (Degryse et al., 2021). Bacterial oxidation is carried out by several specific soil taxa, i.e., the genus *Thiobacillus* (Germida and Janzen, 1993) and *Betaproteobacteria* (Tourna et al., 2014). On the other hand, some other soil microorganisms, mainly fungi, are adversely affected by elemental sulfur (Williams and Cooper, 2004; Massalimov et al., 2012). Elemental sulfur oxidation is dependent on soil water potential, temperature, aeration (Germida and Janzen, 1993), hydrophobicity of its particles, and their size (Steudel, 2003). Oxidation rate depends indirectly on elemental sulfur particle size (Watkinson and Blair, 1993), therefore, fine-formulated (micronized, nanosized) elemental sulfur is used for accelerated conversion to sulfates and nutrient availability (Chapman 1989; Soltanaeva et al., 2018), improved plant nutrition efficiency (Matamwa et al., 2018; Soltanaeva et al., 2018), enhanced alleviation of metalloid toxicity (Dixit et al., 2015), soil pH modulation (Hu et al., 2007; Almutairi et al., 2017), and plant pests control (Gadino et al., 2011). Crushing, ball milling (Hegedüs et al., 2018; Lonkar et al., 2018), or sonication (Raghavan et al., 2018) are methods used to manufacture micro-/nanosized elemental sulfur, which is sometimes further combined with other types of nanomaterials.

Elemental sulfur combined with carbon nanomaterials (e.g. activated carbon, GO) brings benefits i.e., in environmental and forestry applications (Yang et al., 2019; Jeon et al., 2020; Huang et al., 2021). The stimulatory effect of GO and elemental sulfur on the specific elemental sulfur-oxidizing microbiome in amended soil and successive enhanced transformation to sulfates could be ascribed from the referred supportive impact of graphite plate on the biofilm development of *Acidithiobacillus thiooxidans* (Méndez Tovar et al., 2019). Studies describing similar effects of co-application of nano- or microsized elemental sulfur and GO on soil quality biological indicators, such as respiration (basal as well as substrate-induced) and soil extracellular enzymes activity, have remained largely overlooked. This work aims to better understand this issue and bridge the knowledge gap in order to evaluate the actual benefits of currently developing usage of nanotechnologies in agriculture (Behl et al., 2022). Furthermore, the novelty of this work lies in the original and previously untested combination of GO and elemental nano-sulfur in a composite product, which was designed as a carrier of sulfur for improved accessibility and liability to oxidation and accelerated transformation into plant available form. It is expected that the composite could provide a more quickly transformable form of elemental sulfur and concurrently would not disadvantageously increase its solubility and risk of losses from excessive solubilization and leaching.

Therefore, keeping in view the above background, a pot experiment was carried out to evaluate the combined effect of GO and elemental nano-sulfur (nano-S) on soil pH, biological properties, and dry plant biomass under controlled conditions (growth chamber with artificial light). We hypothesized that:

- GO application would enhance soil respiration due to its function as an oxidative agent.
- Nano-S as an oxidizable substrate would suppress soil respiration, moreover, it could counteract and mitigate the GO-derived effect on organic carbon oxidation.
- However, the effect of both amendments on plant biomass yield may depend on the dose and combination of GO and S.

## 2 Material and methods

### 2.1 Sources and preparation of materials

Nanoparticles of elemental sulfur in water dispersion were purchased from US Research Nanomaterials, Inc (Houston, TX, USA). The method of GO synthesis has been previously described by (Đurović et al., 2022). The nanocomposite of GO with nano-S was synthesized by the following procedure: 25 mL of GO (2 g·L^−1^) was mixed with 25 mL of nano-S (100 g·L^−1^) for high dose and 2.5 mL of nano-S (100 g·L^−1^) for low dose as described in (Hammerschmiedt et al., 2022).

### 2.2 Pot experiment settings

The present pot experiment was carried out under controlled conditions in a growth chamber Climacell EVO (BMT, Czech Republic). The experimental soil consisted of topsoil from a rural area near the town of Troubsko, Czech Republic (49°10’28” N, 16°29’32” E). The collected soil was sieved through a 2.0 mm sieve, and mixed with fine quartz sand (0.1-1.0 mm) in a ratio of 1:1, w/w. The properties of the used silty clay loam (Haplic Luvisol) are stated in (**Table 1**).

**Table 1:** The properties of topsoil used for the pot substrate preparation.

Parameter	Value	Unit	Parameter	Value	Unit
pH(CaCl_2_)	7.29	–	C/N	8.77	–
TC	14.00	g·kg^-1^	S	145	mg·kg^-1^
TN	1.60	g·kg^-1^	P	97	mg·kg^-1^
N_mineral_	62.84	mg·kg^-1^	K	231	mg·kg^-1^
N-NO_3_	56.80	mg·kg^-1^	Ca	3259	mg·kg^-1^
N-NH_4_	6.04	mg·kg^-1^	Mg	236	mg·kg^-1^

pH(CaCl_2_) was determined according to ISO 10390:2005; TC and TN were determined using the Vario Macro Cube (Elementar Analysensysteme GmbH, Langenselbold, Germany); N-NO_3_, N-NH_4_ were determined according to ISO 15476:2009; N_mineral_ is a sum of N-NO_3_ and N-NH_4_ content; C/N was calculated from TC and TN; S, P, K, Ca, Mg were determined according to ISO 15178:2000, ISO 14869-3:2017, and ISO 13196:2013.

Pots of 1 L volume (three replicates per variant) were filled with 1 kg of experimental soil. The control soil variant was not amended, the soil of other variants was mixed in the whole volume with GO and nano-S at various doses and combinations displayed in (**Table 2**). The doses of graphene oxide were estimated as a compromise between the dosing reported by Anjum et al. (2014) and Forstner et al. (2019).

**Table 2:** The experimental variants used in this study.

Abbreviation	Name	Amendment and dose	Replicates
–	Control	–	4
GO	GO	GO 10 mg·kg^-1^ of soil	4
S1GO	Low nano-S + GO	nano-S 0.05 g·kg^-1^ + GO 10 mg·kg^-1^ of soil	4
S2GO	High nano-S + GO	nano-S 0.5 g·kg^-1^ + GO 10 mg·kg^-1^ of soil	4
S1	Low nano-S	nano-S 0.05 g·kg^-1^ of soil	4
S2	High nano-S	nano-S 0.5 g·kg^-1^ of soil	4

The pot experiment with lettuce (*Lactuca sativa* L. var. *capitata* L.) cv. Smaragd was conducted over a period of 8 weeks, during which the following conditions were ensured: full-spectrum LED lighting, intensity 370 µmol·m^−2^·s^−1^; photoperiod 12 h; temperature 18/22°C night/day; and relative air humidity 70%, soil moisture 60% of water holding capacity. Lettuce seeds were germinated for two days on filter paper, then four seeds were sown in each pot to a depth of approximately 2 mm. After sowing, each pot was watered with 100 ml of demineralized water. The 10-day-old seedling was reduced to only one per pot. At the end of the experiment (56 days after sowing), the plants were harvested at ground level, and the roots were removed from the soil and washed with demineralized water. Aboveground biomass (AGB) and roots were air-dried at 60°C to constant weight in a laboratory oven to determine the dry biomass of AGB and roots (AGB_dry and Root_dry) by weighing on laboratory scales (n = 3). A mixed soil sample was also taken from each pot.

### 2.3 Methods for soil properties determination

The following soil properties were evaluated following standard methods, such as pH in CaCl_2_ (ISO_10390, 2005), n = 6; dehydrogenase activity (DHA) (Casida et al., 1964), n = 24; soil basal respiration (BR) and substrate-induced respiration (Campbell et al., 2003): Glc_SIR, Pro_SIR, Tre_SIR, NAG_SIR, Ala_SIR, Man_SIR, n = 12; enzyme activities (ISO_20130, 2018): arylsulfatase, urease, phosphatase, N-acetyl-β-D-glucosaminidase, β-glucosidase, n = 18.

### 2.4 Statistical analyses

Statistical analyses were carried out using program R, version 3.6.1. (R Core Team, 2020). PCA was performed to characterize the relationship between soil properties and dependence on the selected treatments. ANOVA type I (sequential) sum of squares was used to test the statistical effect of the selected treatment on the soil properties. For detecting the statistically significant difference after ANOVA, the Tukey’s honest significant difference (HSD) test at a significance level of 0.05 was employed. Factor level means were determined by using treatment contrast. Besides, the Shapiro-Wilk test for the verification of normality and Levene’s test for the verification of homogeneity of variances were also performed at a significance level of 0.05. Pearson correlation coefficient was used to determine the linear correlation among soil properties.

## 3 Results

### 3.1 Effect of graphene oxide, nano-sulfur, and their combination on soil respiration and enzymes

**Figure 1:**
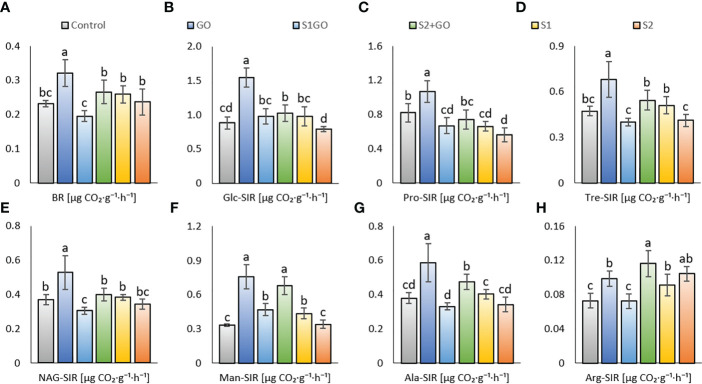
Basal respiration **(A)** and respiration induced by D-glucose **(B)**, protocatechuic acid **(C)**, D-trehalose **(D)**, N-acetyl- β -D-glucosamine **(E)**, D-mannose **(F)**, L-alanine **(G)**, and L-arginine **(H)** in the soil amended with GO, nano-S, and their combination. Mean values (n = 12) are displayed with error bars (standard deviation). Different letters indicate statistically significant differences between variants at p ≤ 0.05.

Determination of basal respiration (BR) and different types of substrate-induced soil respiration (SIR) provided the greatest variability among the tested experimental variants: inducing substrates D-glucose, protocatechuic acid, D-trehalose, N-acetyl-β-D-glucosamine, D-mannose, L-alanine, and L-arginine. Only the variant GO showed a significant increase in BR, Tre_SIR, and NAG_SIR, compared to the Control (**Figures 1A, D, E**). Glc_SIR, Man_SIR, Ala_SIR, and Arg_SIR (**Figures 1B, F, G, H**) were significantly increased in variants GO and S2GO compared to Control, whereas a single amended high dose of nano-S (variant S2) decreased Pro_SIR (**Figure 1C**).

Apparent soil respiration types by added GO were found to be mitigated by the low dose of nano-S, albeit not by the high dose. Therefore, values of BR, Glc_SIR, Tre_SIR, Ala_SIR, and Arg_SIR were comparable between the Control and the variant S1GO (**Figures 1A, B, D, G, H**), whereas Man_SIR, Ala_SIR, and Arg_SIR were significantly increased in S2GO compared to both the Control and S1GO (**Figures 1F, G, H**). Further, a single application of a high dose of nano-S had a negative impact on sugar-induced respirations (Glc_SIR, Tre_SIR, and Man_SIR) as compared to the variants with a low nano-S dose. In general, a single application of GO was beneficial for all types of soil respiration, whereas amendment of GO combined with nano-S enhanced only Man_SIR, Ala_SIR, and Arg_SIR respiration at high nano-S dose. Nano-S amendment did not significantly affect respiration (except for increased Arg_SIR – both variants S1, S2 – and Man_SIR, variant S1), nor did it have a significant negative effect (Pro_SIR) and the benefit of the high nano-S dose was less beneficial.

**Figure 2:**
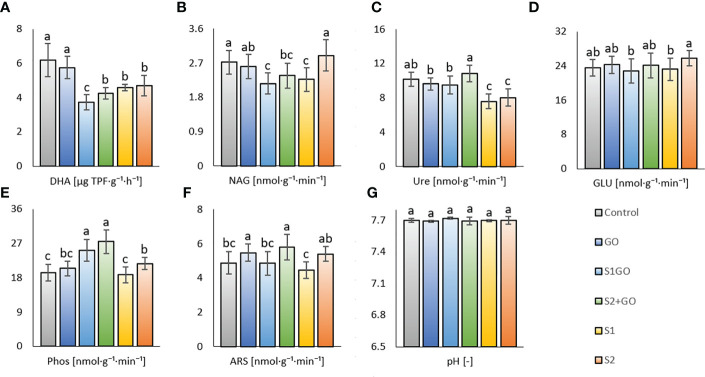
Dehydrogenase **(A)** (n=24), N-acetyl- β -D-glucosaminidase **(B)**, urease **(C)**, β -glucosidase **(D)**, phosphatase **(E)** arylsulfatase **(F)**, and activities (n=18) and pH **(G)** (n=6) in the soil amended with GO, nano-S, and their combination. Mean values are displayed with error bars = standard deviation. Different letters indicate statistically significant differences between variants at p ≤ 0.05.

Beneficial to partially (significantly) positive effect of single GO application was also detected *via* determination of soil enzyme activities; DHA, NAG, Ure, GLU, and Phos activities of variant GO were comparable to the Control and ARS was significantly increased (**Figures 2A–F**). Similarly, significant positive effects of High nano-S + GO on Phos and ARS (compared to Control) were revealed (**Figure 2E, F**), albeit DHA and N-acetyl-β-D-glucosaminidase NAG were affected significantly negatively by nano-S + GO at both doses (**Figures 2A, B2**). The benefit of low nano-S + GO was again weaker than high nano-S + GO, showing values of Ure, GLU, and ARS, comparable to the Control, the only significant increment (compared to the Control) was found for Phos (**Figure 2C, F**). Whereas a single amendment of the high nano-S dose was less beneficial to soil respiration (Glc_SIR, Tre_SIR, and Man_SIR), activities of NAG, GLU, Phos, and ARS, of this variant (S2) showed (compared to variant S1) significant increment, **Figures 2B, D–F**. In general, any variant amended with nano-S showed an adverse effect on DHA; moreover, single-applied nano-S at both doses mitigated Ure activity. While nano-S + GO (both S1GO and S2GO) enhanced the activity of Phos.

### 3.2 Effect of graphene oxide, nano-sulfur, and their combination on soil pH and plant biomass

**Figure 3:**
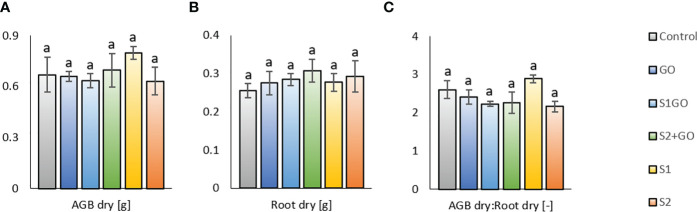
Dry aboveground **(A)**, root **(B)** biomass, and their ratio AGB_Root **(C)** for lettuce grown in the soil amended with GO, nano-S, and their combination. Mean values (n = 3) are displayed with error bars = standard deviation. Different letters indicate statistically significant differences between variants at p ≤ 0.05.

The described values of soil respiration and enzyme activities in all experimental variants were likely negligibly influenced (**Figure 2G**). The response of soil parameters to the application of GO and Nano-S was not significantly related to the final values of aboveground dry matter (AGB) and root biomass of lettuce, which were comparable for all variants including the Control variant (**Figures 3A, B**). Some markable results of the determined biomass properties were the relatively highest average dry AGB value of variant S1 and the relatively lowest average root biomass weight in the Control. The outcome of these findings was the highest AGB_Root biomass ratio in the variant S1 (and in the Control as well – this indicates improved shoot growth), compared to the other variants, i.e., the variant S1GO, which in contrast stimulated the growth and biomass of roots, instead of shoots (**Figure 3C**).

## 4 Discussion

### 4.1 Effect of graphene oxide, nano-sulfur, and their combination on soil respiration and enzymes

It was referred that GO in soil may harm microorganisms by penetrating cell walls and extracting phospholipids (Tu et al., 2013), as well as by degreasing abundances of different functional microbial groups associated with the processes of nutrient transformation and respiration (Du et al., 2020). The most influenced soil properties in the experiment were basal and substrate-induced respiration, which (with the exception of Man_SIR and Ala_ SIR) were significantly higher in the soil enriched with GO compared to all other variants. These findings did not prove an adverse effect of GO but corroborated our hypothesis (i). GO can be reversibly reduced and oxidized because it enhances electron transfer (Pan et al., 2017). Apart from several studies which referred to the adverse effect of GO on soil microbiome (Gurunathan et al., 2012; Du et al., 2015; Xie et al., 2016), the positive effect on microbial growth and activity is assumed due to reported improved delivery of macro- and micronutrients *via* adsorption (Kabiri et al., 2017; Navarro et al., 2020). Nevertheless, few studies described the GO effect on microbial respiration, e.g. addition of GO (up to 60 mg·L^-1^) increased the oxygen uptake rate coefficient of municipal landfill leachate bacterial cultures (Jamialahmadi et al., 2018) and aerobic bacteria isolated from various environments, including soil, were able to increase GO reduction under respiration (Salas et al., 2010; Chouhan et al., 2016). However, our previous study (Hammerschmiedt et al., 2022) showed contrasting results of various types of microbial respiration in lettuce-planted soil under illumination with color (blue+red) light (20 klx), where the GO amended variant showed comparable or lower (Glc_SIR) values than the Control. Due to the relation between illumination (intensity and quality) and plant physiology (Gouinguene and Turlings, 2002; Hee-Sun Kook, 2013), both stimulation, and composition, as well as activity of the microbial community in the rhizosphere, were reported in several studies e.g., blue color light stimulation of multiplication of moldy fungi (Bonomi et al., 2012; Borowiak et al., 2019).

High nano-S + GO (variant S2GO) had a positive effect on these particular types of soil respiration (Glc_SIR, Man_SIR, Ala_SIR, and Arg_SIR) in comparison to the Control, whereas single soil application of high nano-S led only to the comparable values (as the Control) of the respective properties (except significantly increased Arg_SIR, **Figures 1B, F–H**). These findings partially corroborated our hypothesis (ii), confirming a negative impact of sole nano-S addition on respiration but a positive effect of a combination of both amendments. As compared to the GO variant or even the Control, it was ascribed that the impact of a low dose of nano-S (variant S1) on the soil respiration was neutral or negative, which was in line with the results of our previous study (Hammerschmiedt et al., 2022). Thus, elemental (nano)sulfur has been reported to attenuate soil aerobic microbial activity and the respiratory capacity of the soil microbiome, as described in a few studies (Kelleher et al., 2017; Zakari et al., 2020). Nevertheless, this study showed a less significant negative effect of nano-S on the GO-associated aerobic carbon mineralization benefits than in the previously conducted pot experiment (Hammerschmiedt et al., 2022), which showed a very strong negative effect of the nano-S + GO. In line with these results, Kelleher et al. (2017) referred to an initial surge in the production of CO_2_ through microbial respiration, which was followed by increased capture of carbon dioxide as elemental sulfur was oxidized to sulfate (Kelleher et al., 2017). The conditions set up in this experiment did not promote complete nano-S-mediated attenuation of GO stimulation. However, significant mitigation of most types of respiration in the variant S2GO and even more in the S1GO variant, compared to the values of variant GO, was evident. We corroborated our hypotheses (ii) and (iii) that nano-S would counteract the GO-mediated beneficial effect of organic carbon oxidation and weaken the stimulation of soil respiration, and that these effects would be dose-dependent. An even higher negative effect of nano-S + GO co-application (compared to both sole amended GO and sole low nano-S) was observed in the variant S1GO for the properties NAG_SIR, Tre_SIR, Ala_SIR, and Arg_SIR (**Figures 1D, E, G, H**), which values were significantly lowered compared to the single-treated variants (GO, S1). We presumed that this feature was caused by a generally decreased degradation activity of soil microbiome in this (S1GO) variant, which was ascribed the lowest value of dehydrogenase (DHA) in comparison to all other variants (**Figure 2A**). Even at the lower nano-S dose in this variant (S1GO), the highly oxygen-dependent aerobic catabolism of particular substrates (e.g., protocatechuic acid) putatively competed with the oxygen-demanding elemental sulfur utilization. These presumptions were supported by the results of the PCA analysis (synergy between dehydrogenase and respirations) and Pearson correlation analysis (**Supplementary Figures 1, 2**), showing a significant (p < 0.001) positive correlation of DHA and Pro_SIR (r = 0.62); further, BR and Tre_SIR, NAG_SIR, and Ala_SIR (r were 0.69, 0.7, 0.72) were also correlated.

As we mentioned in the previous paragraph, dehydrogenase activity (DHA), which indicates the ability of soil microbiome to degrade soil organic matter (SOM), was decreased by the application of nano-S at both doses as compared to the Control and GO variant, **Figure 2A**. N-acetyl-β-D-glucosaminidase (NAG), an enzyme involved in the degradation of main fungal cell wall polymer chitin, also exerted a decrease in most nano-S-treated variants (S1GO, S2GO, and S1 - except for S2) compared to Control (**Figure 2B**). These results again contrast with a previous study (Hammerschmiedt et al., 2022), in which the addition of a very high dose of Nano-S (1 g S. kg^-1^) stimulated both dehydrogenase and NAG activities (compared with the Control). The positive effect of high dose nano-S on the preservation of NAG activity (of variant S2, comparable to the Control) in this current experiment allowed us to presume that the efficient elemental sulfur stimulation of fungal biomass multiplication (due to the fungal involvement in elemental sulfur oxidation (Germida and Janzen, 1993)) occurs at higher application levels. This proved hypothesis (iii). The results of the respective previous study corresponded to the referred beneficial effect of waste elemental sulfur application on soil DHA in unsown arable soil (Tabak et al., 2020). On the other hand, a dose of 50 mg.kg^-1^ of elemental sulfur added to alkaline S-deficient soil did not affect DHA (Malik et al., 2021), similarly observed in this study. We further assumed that the activity of NAG could be enhanced in the first experiment due to the putative blue color light stimulation of moldy fungi growth (Borowiak et al., 2019), and subsequent higher access of residual fungal biomass in soil. Contrary to the effect of nano-S, GO amendment to soil helped to preserve values of DHA and NAG (as well as other enzymes – urease, β-glucosidase, phosphatase) comparable with the Control, which was in contrast with the referred detrimental effect of GO (Chung et al., 2015), but close to the opposite reports of a beneficial effect of graphene-based nanomaterials on soil enzymatic activities (Ren et al., 2015). The other study (Rong et al., 2017) showed that the positive or negative effect of either graphene or GO on microbial enzymes was vastly dependent on the composition of the soil microbial community. Arylsulfatase activity was enhanced by the single soil application of GO (compared to the Control) Arylsulfatase, as the only one from determined enzymes. had enhanced activity by the single soil application of GO (compared to the Control). We attributed this result to the reported positive role of GO in sulfur oxidation and mineralization, as it was referred (Rong et al., 2017), and to the beneficial general effect of GO on distinct soil microbiota and their activity (Rong et al., 2017). Nevertheless, GO-promoted ARS activity was again discordant to the previous study (Hammerschmiedt et al., 2022) and the role of different illumination in the soil microbial community composition (as reported by (Carvalho and Castillo, 2018)) and their related enzyme activities could be considered. As well as variant GO, high nano-S + GO increased the activity of ARS in comparison to the Control (**Figure 2F**); therefore, we deduced that GO mitigation of enzyme activity (Chung et al., 2015) did not overwhelm the positive effect of elemental sulfur on ARS (Malik et al., 2021).

The only variant with significantly increased Ure activity (compared to the Control) was high nano-S + GO (S2GO), while both doses of nano-S applied alone (S1, S2) to the soil showed significantly decrease Ure activity (as compared to the Control). We assumed that next to the oxidative mineralization of nano-S, the reductive transformation to sulfides and hydrogen sulfide could also occur in the soil. Considering the referred inhibition of nitrification activity by elevated sulfide levels (Joye and Hollibaugh, 1995) we ascribed that higher access of nano-S in soil (S1, S2) might have decreased the Ure activity.

No significant change in β-glucosidase activity was observed in any of the altered variants compared to the Control (**Figure 2D**). A significant decline in GLU activity was detected for the low-dose nano-S-amended variants (S1GO and S1) as compared to the sole high nano-S amended variant (S2). This feature was consistent with reports of increased GLU in soils receiving higher levels of elemental sulfur (Ye et al., 2011). The most significant enzyme response to the amendment of GO (in combination with a low or high dose of nano-S) showed Phos, both GO variants with nano-S (irrespective of dose) increased Phos as compared to the Control. We related these results to the effect of elemental sulfur – there was reported higher Phos activity promoted by sulfur fertilization at the background of NPK (Godlewska, 2018) – and to the putatively higher retention of soil phosphate content due to interaction with added GO. Absorption properties of GO were reported to be beneficial for phosphate loading on the surfaces of the nanoparticles (Kabiri et al., 2020), and we ascribed from this decreased leaching and losses of phosphorus (P) and thus, its higher P accessibility to transformation (enzyme-catalyzed). A general positive effect of GO on the increased availability of various nutrients to transformation processes may be ascribed from synergy (PCA biplot, **Supplementary Figure 1**) and significant (p < 0.001) positive correlation of Phos and Ure, ARS (r were 0.42 and 0.52, respectively), while enhanced SOM degradation could increase P losses as assumable from the negative correlation of Phos and DHA (r = -0.47) and antagonism (PCA biplot), **Figures A1**, **A2**. Further, we hypothesized (i.): if the sulfur transformation (locally in the rhizosphere) takes place under oxidative conditions, the soil would tend to slightly lower pH and improve phosphate dissolution, which would further increase its availability and, subsequently, Phos activity. A significant antagonism (PCA biplot, **Supplementary Figure 1**) and negative correlation between pH and Phos was detected (r = -0.53, p < 0.001) (**Supplementary Figure 2**).

### 4.2 Effect of graphene oxide, nano-sulfur, and their combination on soil pH and plant biomass

However, no significant differences in soil pH values were observed between the variants (**Figure 2G**). Neither the final values of lettuce dry aboveground and root biomass, which were comparable between all variants including the Control (**Figures 3A, B**), displayed any significant effect of presumed and detected differences in the soil biological properties, determined by the differing diversity of soil microbial community. Nevertheless, the relatively highest average dry AGB value of variant S1 and the relatively lowest average root biomass in the Control were coupled with the highest AGB_Root biomass ratio in variant S1 (and in the Control), compared to the other variants. These results indicated that the sulfur uptake preferentially contributed to the growth of aboveground parts of lettuce. It agreed with the research findings based on a greenhouse experiment, in which the application of elemental sulfur (570 mg.kg^−1^) significantly increased stem diameter, plant height, shoot weight, and sulfate uptake by maize plants (Pourbabaee et al., 2020). Another study reported that elemental sulfur amended to the soil at a rate of up to 50 mmol.kg^-1^ elemental sulfur also led to a higher concentration of sulfur in the shoots than in the roots (Cui and Wang, 2005).

On the contrary, the variant S1GO (low nano-S + GO) stimulated the growth and biomass of roots, instead of shoots (**Figure 3C**). It was referred that very high (> 400 mg.L^−1^ GO) amendment of GO to soil significantly increased root biomass and length (Anjum et al., 2014). However, our result seemed closer to the study of (Xiao et al., 2022), which reported little effect of GO exposure at doses 10 and 100 mg.L^-1^ on plant growth. Increased nutrient availability to lettuce plants, derived by GO and its physicochemical properties as reported (Lahiani et al., 2015; Kabiri et al., 2017; Juarez-Maldonado et al., 2019; Carneiro et al., 2022), could have been one of the possible reasons. Another mechanism of beneficial interaction of GO and elemental sulfur could have been a positive effect on soil water retention as referred to by (Zhao et al., 2020; Zhao et al., 2022). In the previously published study by (Hammerschmiedt et al., 2022), in which the lettuce plants were illuminated by color (blue+red) light instead of artificial white light, the plant biomass yield responded to GO and co-applied S^0^ + GO differently, showing the significantly higher AGB values than other amended soil variants. These significant differences could be ascribed to the presumed contrasting plant physiology and qualitative properties of plant biomass, which were not determined, and coupled with the significantly weaker positive impact of GO (applied solely or with S^0^) on soil microbial properties, namely basal and other types of soil respiration. Thus, concerning the findings of this research, as well as the previous one (Hammerschmiedt et al., 2022), it can be concluded that the different types of supplements exerted contradictory effects on soil biological properties and plant growth in a similar way, as found, for example, for GO in recent studies (Fattahi et al., 2022).

## 5 Conclusions

GO applied on its own exerted the most positive effect on soil respiration, which was still significant in the combination of high nano-S + GO. While low nano-S + GO negatively affected some types of soil respiration (NAG_SIR, Tre_SIR, Ala_SIR, and Arg_SIR). We verified our hypothesis that elemental nano-S would probably counteract the GO-mediated effect of organic carbon oxidation. The benefit of GO was detectable *via* the determination of soil enzyme activities: GO on its own enhanced ARS, and GO + high nano-S dose enhanced ARS, Ure, and Phos. Several contrasts were found between the results of this experiment and the previously carried-out pot trial, performed with nanosized (microsized) elemental sulfur and GO in soil sown with lettuce and illuminated by color (blue+red) light instead of artificial white light. This difference in the experimental conditions was the most noticeable in the contrasting effect of GO (applied on its own or with S^0^) on plant biomass quantity (and presumably also quality) and concurrently on plant growth-associated soil biological properties, namely respiration indicators.

## Data availability statement

The original contributions presented in the study are included in the article/**Supplementary Material**. Further inquiries can be directed to the corresponding authors.

## Author contributions.

TH was involved in conceptualization, data curation, and writing the original draft. JH was involved in data curation, investigation, and writing, namely review and editing. RZ was involved in software and methodology. AK was involved in formal analysis, investigation, and software. PS was involved in conceptualization, methodology, and writing, namely review and editing. ZB was involved in software and writing, namely review and editing. LR was involved in formal analysis, supervision, and writing, namely review and editing. AM was involved in formal analysis and writing, namely review and editing. OM was involved in data curation and validation. MB was involved in conceptualization, formal analysis, funding acquisition, supervision, and writing, namely review and editing. All authors contributed to the article and approved the submitted version
