# Supplementary material for: The combined effect of graphene oxide and elemental nano-sulfur on soil biological properties and lettuce plant biomass
Source: Front Plant Sci. 2023 Mar 14;14:1057133. doi: 10.3389/fpls.2023.1057133 (PMC10043190; doi:10.3389/fpls.2023.1057133)
Supplement: Supplementary file 1 [file DataSheet_1.docx]

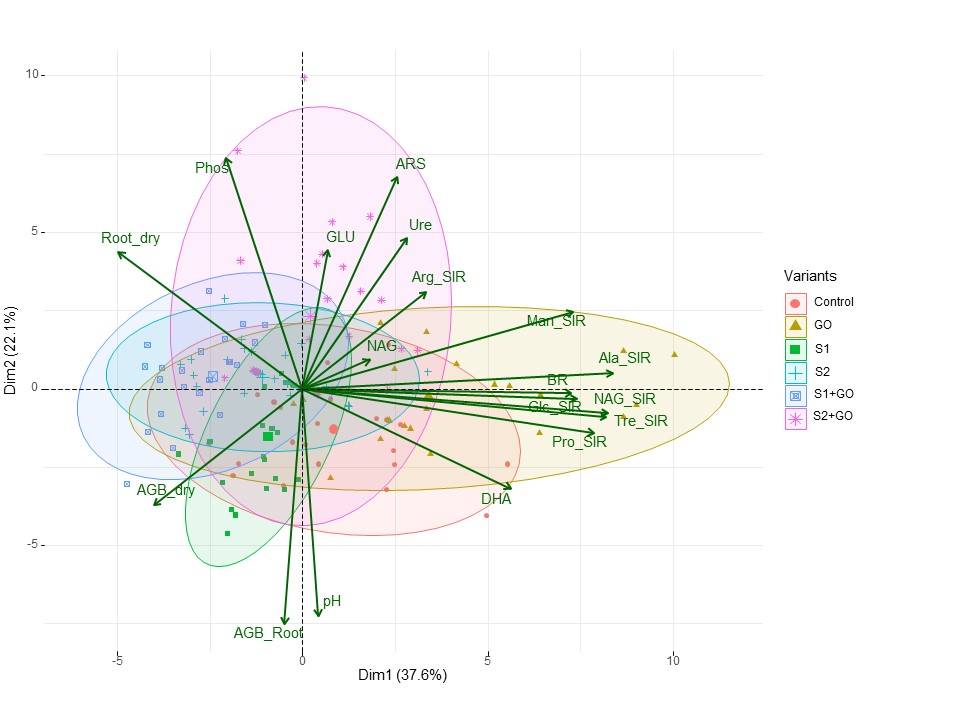


**Supplementary Figure 1.** PCA biplot analyses of results from plant and soil properties determination


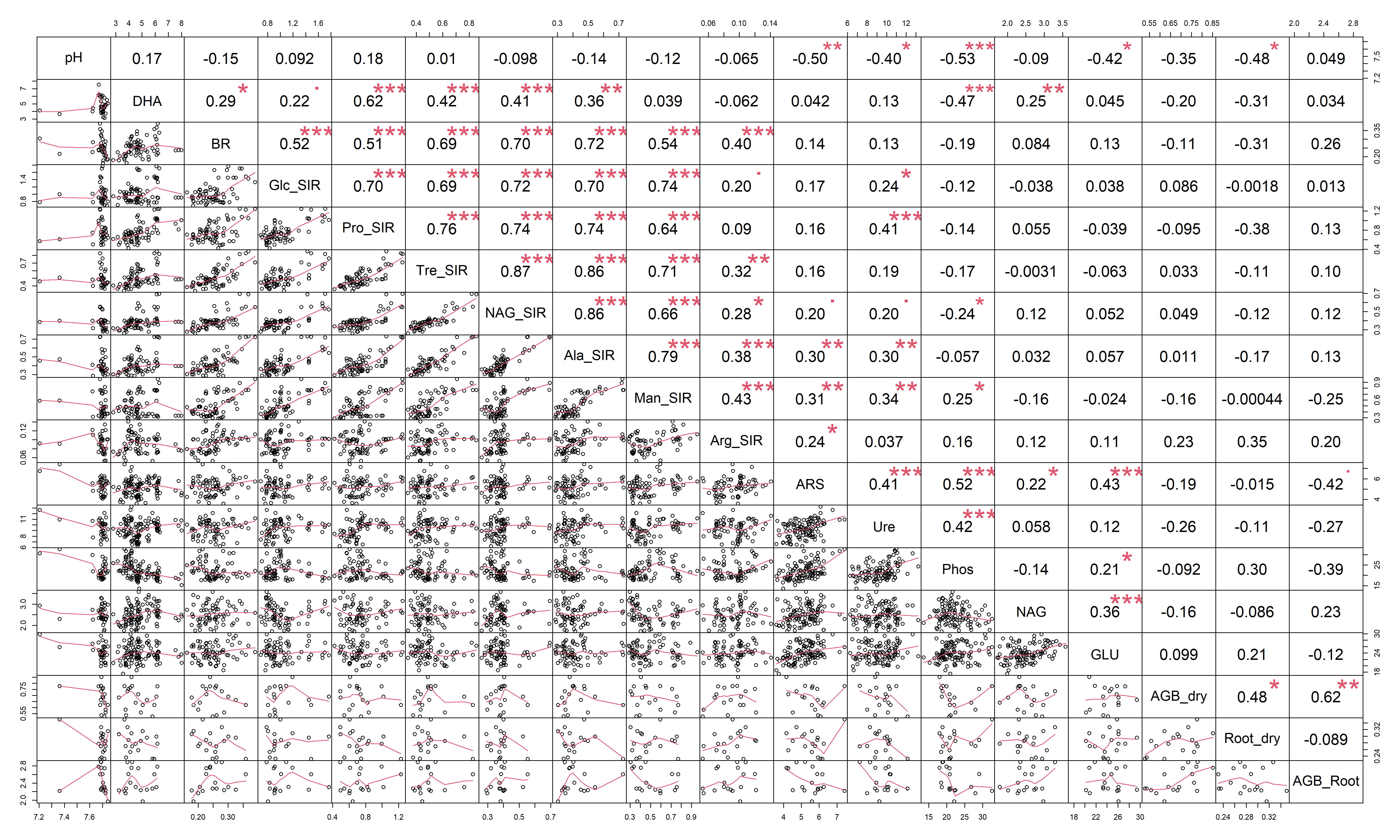
 **Supplementary Figure 2.** Pearson correlation matrix of results from plant and soil properties determination

The stars indicate a level of significance in statistical difference between the variables: * for p≤0.05, ** for p≤0.01, *** for p≤0.001.
